# Human Brain Organoids as Models for Central Nervous System Viral Infection

**DOI:** 10.3390/v14030634

**Published:** 2022-03-18

**Authors:** Josse A. Depla, Lance A. Mulder, Renata Vieira de Sá, Morgane Wartel, Adithya Sridhar, Melvin M. Evers, Katja C. Wolthers, Dasja Pajkrt

**Affiliations:** 1 OrganoVIR Labs, Department of Medical Microbiology, Amsterdam UMC Location Academic Medical Center, Amsterdam Institute for Infection and Immunity, University of Amsterdam, Meibergdreef 9, 1105 AZ Amsterdam, The Netherlands; l.a.mulder@amsterdamumc.nl (L.A.M.); a.sridhar@amsterdamumc.nl (A.S.); k.c.wolthers@amsterdamumc.nl (K.C.W.); d.pajkrt@amsterdamumc.nl (D.P.); 2 Department of Pediatric Infectious Diseases, Emma Children’s Hospital, Amsterdam UMC Location Academic Medical Center, University of Amsterdam, Meibergdreef 9, 1105 AZ Amsterdam, The Netherlands; 3 UniQure Biopharma B.V., Department of Research & Development, Paasheuvelweg 25A, 1105 BE Amsterdam, The Netherlands; r.vieiradesa@amsterdamumc.nl (R.V.d.S.); m.wartel@uniqure.com (M.W.); m.evers@uniqure.com (M.M.E.)

**Keywords:** brain organoids, cerebral organoids, Zika virus (ZIKV), severe acute respiratory syndrome coronavirus 2 (SARS-CoV-2), herpes simplex virus 1 (HSV1), human cytomegalovirus (HCMV), Dengue virus (DENV), Measles virus (MeV), La crosse virus (LACV), Japanese encephalitis virus (JEV)

## Abstract

Pathogenesis of viral infections of the central nervous system (CNS) is poorly understood, and this is partly due to the limitations of currently used preclinical models. Brain organoid models can overcome some of these limitations, as they are generated from human derived stem cells, differentiated in three dimensions (3D), and can mimic human neurodevelopmental characteristics. Therefore, brain organoids have been increasingly used as brain models in research on various viruses, such as Zika virus, severe acute respiratory syndrome coronavirus 2, human cytomegalovirus, and herpes simplex virus. Brain organoids allow for the study of viral tropism, the effect of infection on organoid function, size, and cytoarchitecture, as well as innate immune response; therefore, they provide valuable insight into the pathogenesis of neurotropic viral infections and testing of antivirals in a physiological model. In this review, we summarize the results of studies on viral CNS infection in brain organoids, and we demonstrate the broad application and benefits of using a human 3D model in virology research. At the same time, we describe the limitations of the studies in brain organoids, such as the heterogeneity in organoid generation protocols and age at infection, which result in differences in results between studies, as well as the lack of microglia and a blood brain barrier.

## 1. Introduction

Viral infections of the central nervous system (CNS) can cause severe morbidity with associated short and long-term sequelae and mortality. Viral CNS infection can lead to inflammation of the meninges lining the brain (meningitis), the brain itself (encephalitis), and the spinal cord (myelitis) [1]. Common viruses to cause CNS disease are herpesviruses, enteroviruses, and arboviruses [2,3,4]. Neurotropic viruses are able to overcome the blood brain barrier to invade the CNS and cause disease through virus-induced cytopathology or neurotoxic antiviral immune responses [1,5]. For most of these viral infections, specific therapies are lacking. Our current understanding of the pathogenesis of CNS viral infections is attributed to work in preclinical models, such as animal models and cell lines. However, the further elucidation of host virus interactions and the development of effective antivirals and vaccines are hampered by the unavailability of suitable human based models and by the bounds to which current preclinical models can accurately recapitulate human disease. The translational power of cell lines is limited by the fact that they are typically grown in a monoculture in two dimensions (2D), and as such, they lack the polarity, cell-to-cell, and cell-extracellular matrix interactions that are found in the complex multicellular microenvironment of the human brain [6]. Additionally, there are differences in gene expression and host pathogen interactions between cells grown in three dimensions (3D) compared to 2D cultures [7,8,9]. Finally, cell lines consist of transformed cells, which may harbor oncogenic gene mutations that can alter viral replication [10]. While 2D cell lines lack complexity, animal models enable the study of infection in a systemic manner. However, human viruses are specific to humans, through co-evolution with their host, so animal models, therefore, have the limitation that they cannot model species-specific differences in viral infection [11]. For instance, there are species-specific differences in the spontaneous reactivation of latent HSV1 infection [12]. For studying viruses that affect neurodevelopment, animals have the limitation that they cannot recapitulate features of human-specific neurodevelopment. For example, outer radial glia (oRG) are only sparsely present in rodents [13], while their expansion in humans is central to human neurodevelopment [14]. Lastly, to be susceptible to viral infection, animal models often require transgenic overexpression of human receptors, such as mice models for SARS-CoV-2 infection, or knock out of immune responses, as is the case for mice models for ZIKV infection [10,15]. Therefore, physiologically relevant human models are crucial for a better understanding of the mechanisms of disease and for the development of effective therapies.

Stem cell-derived brain organoids may overcome some of these limitations, as they are generated from human induced pluripotent stem cells (iPSCs) or human embryonic stem cells (ESCs). Human brain organoids are cell cultures, grown in 3D, that contain a mixture of CNS cell types and recapitulate some features of human brain development [13]. A large variety of available protocols describe either the generation of an organoid that models the whole brain through non-guided differentiation, known as cerebral organoids, or the formation of specific regions of the human brain, such as the forebrain, hindbrain, telencephalon, and choroid plexus (ChP) organoids [16,17]. Both methods of organoid generation have been deployed in the field of virology [18]. Brain organoid models are a suitable reflection of the human brain, as their development parallels the development of the human brain, with different cell types arising over the course of organoid maturation [17]. Within brain organoids, fluid-filled lumina are formed that resemble ventricles, which are surrounded by a layer of proliferating neural precursor cells (NPCs, describing both neural stem cells and neural progenitor cells), similar to a developing neural tube in vivo. Just as in the human cortex, the cells within brain organoids migrate radially over time, through the subventricular zone to the cortical plate, while differentiating into neurons [13,17,19]. Besides NPCs and neurons, glial cells, such as astrocytes, ependymal cells, and oligodendrocytes, are also present within the organoids, albeit at later maturation stages, similar to the in vivo situation [20,21]. In general, brain organoid composition is limited to CNS cell types of ectodermal origin and lacks cell types of non-ectodermal origin, such as microglia, the resident immune cells of the CNS, [22,23] and vascular cells. Brain organoids, therefore, lack circulation and fail to model the blood-brain barrier (BBB) [24]. The lack of these cell types limits, to a certain extent, the study of viral immune response and neuroinvasion in organoid studies. However, recent protocols describe strategies to incorporate microglia in brain organoids [22,25,26], and many publications focus on integrating BBB components in cerebral organoids in co-culture models [27,28,29,30]. Additional limitations of brain organoids should be considered, such as their fetal identity and the lack of nutrient and oxygen delivery to the center of the organoids, which leads to the formation of a necrotic core [20]. 

Recently, other reviews have been published discussing the potential and methodology of brain organoids for virology research [12,18,31,32,33,34]. These reviews focus on the application of brain organoids and other human stem cell models for a specific virus, multiple viruses [12,31,32,33,34], or on the types of brain organoid generation protocols used for virology [18]. In this review, we include all research on viral CNS infections using iPSC and ESC human brain organoids. We summarize the outcomes of those studies and demonstrate how brain organoids have been used to study multiple neurotropic viruses. We emphasize the variation in organoid generation protocols and the organoid age at the time of infection. We conclude that the human origin and the 3D cytoarchitecture, consisting of multiple cell types with physiologically relevant cellular identities, make brain organoids interesting models that can give insights into the susceptibility of the CNS, pathogenesis of CNS disease, immune response, and potential antiviral agents against neurotropic viruses. Furthermore, we describe limitations of the studies in brain organoids, such as the variation in results between studies, in part due to variation in maturation states of the organoids at the time of infection.

## 2. Methods

**Figure 1:**
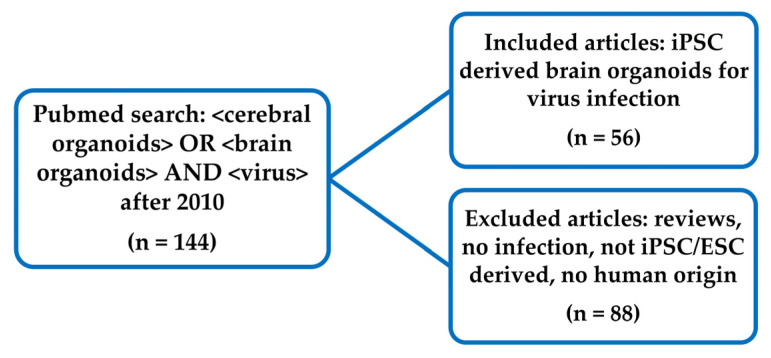
Flow diagram of included articles. Articles were included that used brain organoids derived from induced pluripotent stem cells (iPSCs) or human embryonic stem cell (ESCs) for studying viral infection.

On the 6th of March 2021, we performed a PubMed search for the terms “<cerebral organoids> OR <brain organoids> AND <virus>” (Figure 1) to include studies using whole brain and region specific brain organoids. Papers prior to 2010 were excluded, as these studies were published before the first protocol on brain organoid generation. Papers describing original research on viruses, using human induced pluripotent stem cells (iPSCs) or human embryonic stem cell (ESCs) derived brain organoids, were included. All included papers are listed in Supplementary Table S1. In this review, we use the term brain organoids as an umbrella term for the different organoid protocols used. We noted which viruses were studied and how many papers studied a specific virus. For each study, we documented the age of the human brain organoid at time of the viral infection, and for viruses that were reported in at least four scientific publications, the median brain organoid age was calculated. Additionally, we noted if the studies were performed on cerebral organoids or brain region-specific organoids. We extracted the research questions that were studied by assessing if papers reported on (1) viral infection of the organoid by reporting on expression or replication of viral genomes, (2) viral receptor expression in the brain organoid, (3) changes to the organoid cellular organization and brain organoid size due to the viral infection, (4) viral tropism, (5) viral infection mediated cytopathic effects, (6) reduction in cellular proliferation, (7) innate immune responses, (8) changes in gene expression profiles, and (9) antiviral testing.

## 3. Results

### 3.1. Viruses and Their Effects Studied in Brain Organoids

**Figure 2:**
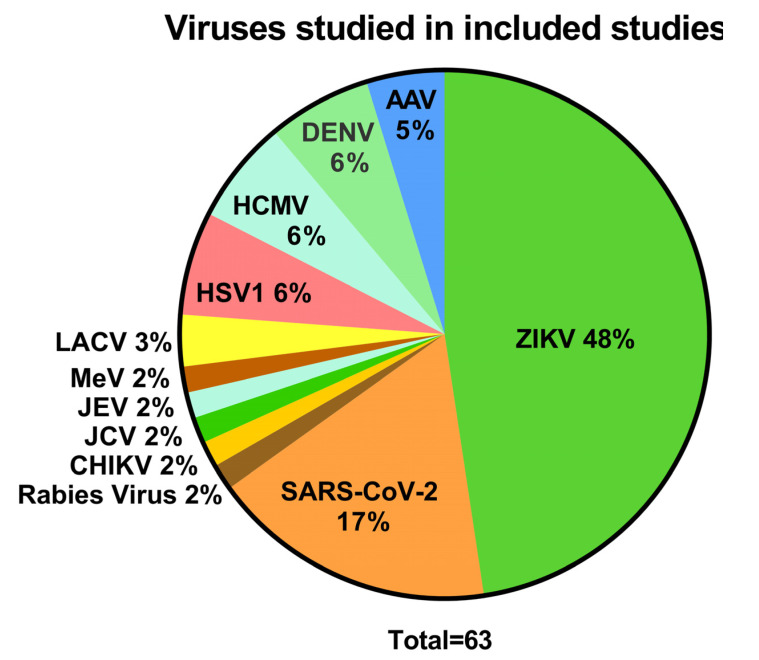
The variety of viruses studied. (Zika virus (ZIKV), severe acute respiratory syndrome coronavirus 2 (SARS-CoV-2), John Cunningham virus (JCV), Chikungunya virus (CHIKV), herpes simplex virus (HSV), human cytomegalovirus (HCMV), dengue virus (DENV), adeno associated virus (AAV), measles virus (MeV), Rabies virus, Japanese encephalitis virus (JEV), and lacrosse virus (LACV)) using human brain organoids. The total number of studies exceeded the number of included papers, as some papers reported on multiple viruses.

To study the outcomes of research on viral CNS infections using human brain organoids, original articles published after 2010, on virus infections in brain organoids, were extracted from PubMed. Among the 56 included articles (Table S1), the majority reported on Zika virus (ZIKV) (30/63, 48%) (Figure 2), followed by severe acute respiratory syndrome coronavirus 2 (SARS-CoV-2) (11/63, 17%), herpes simplex virus 1 (HSV1) (4/63, 6%), human cytomegalovirus (HCMV) (4/63, 6%), Dengue virus (DENV) (4/63, 6%), La crosse virus (LACV) (3/63, 3%), and Adeno associated virus (AAV) (3/63, 5%). Studies on Japanese encephalitis virus (JEV), La crosse virus (LACV), measles virus (MeV), John Cunningham virus (JCV), Chikungunya virus (CHIKV), and rabies virus infection of the CNS were only reported once, respectively. Other viruses that are common causes of CNS disease were not studied using brain organoids, such as enteroviruses, varicella zoster virus, and West Nile virus.

**Figure 3:**
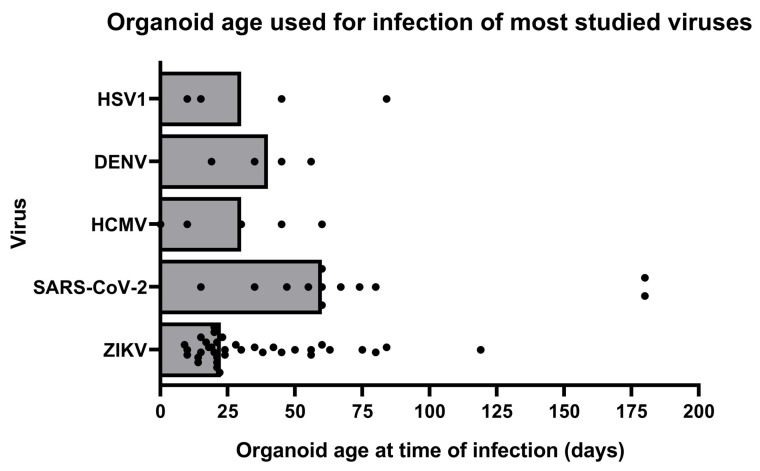
Brain organoid age, at time of infection, in studies on infection of HCMV, HSV1, SARS-CoV-2, and ZIKV (in days). Bars represent median organoid age, and each dot is a single study on the respective virus.

To investigate the versatility of brain organoids, we scored the applications for which brain organoids were used. Across all studied viruses, brain organoids have mainly been used to investigate the susceptibility of the CNS to viral infection (90% of studies), the viral tropism (54% of studies), the cytopathic effect of infection (44% of studies), and the effect of infection on organoid organization and size (59% of studies) (Table 1). These results are discussed in Table 1 below. To a lesser extent, the brain organoids have been applied to study viral receptor expression in the CNS, the effect of infection on cellular proliferation (19% of studies), the type of innate immune response in the organoid (27% of studies), changes in gene expression profiles after infection (22% of studies), and the effectiveness of antiviral drugs (29% of studies). The brain organoids were used as models for viral infection at different maturation stages, which influenced their cellular composition and gene expression. Human brain organoids were infected with SARS-CoV-2 at a median organoid age of 60 days, which was at a later organoid age compared to infections with ZIKV (median age of 23 days), HCMV (median age 30 days), HSV1 (median age 30 days), or Dengue virus (median age 40 days) (Figure 3). Between studies on the same virus, a high variation of organoid ages used for infection was observed. For instance, studies on ZIKV were performed on brain organoids ranging from 9 to 119 days in culture. Besides the large differences in organoid age at time of infection, studies also varied in the type of brain organoid that was used to study a specific virus. A total of 67% of the studies were performed on cerebral organoids that model the whole brain, while 33% used brain-region specific organoids. To demonstrate the broad application of brain organoids in CNS virology, we will next summarize the main results for each studied virus, with a focus on the subjects summarized in Table 1.

### 3.2. Outcomes of Virus Infections in Brain Organoids

In our search, we found 52 articles on multiple neurotropic viruses, which provide insights into the susceptibility and immune responses of the human brain against virus infections, as well as possible antiviral strategies. Therefore, we summarized findings on viral infection and pathogenesis in brain organoids per individual virus and in Table 2. 

#### 3.2.1. ZIKV

ZIKV is an enveloped, single stranded RNA virus from the *Flaviviridae* family. ZIKV has gained increased interest after an outbreak in Brazil in 2015, where the infection of pregnant women was associated with microcephaly—a severe birth defect as a result of underdevelopment of the cerebral cortex—in their newborns [35,36].. Given the urgency of this public health concern, it was a high priority to understand the pathogenesis of ZIKV-induced microcephaly in order to develop an effective treatment. In that regard, the focus of ZIKV research, using brain organoids, was mainly on understanding the effects of ZIKV infection on the brain organoid organization and brain organoid size (27/30 publications, 90%; Table 1), ZIKV tropism (14/30 publications, 47%), cytopathic effect (17/30 publications, 57%), and on the testing of therapeutics (12/30 publications, 40%). 

Overall, infection of brain organoids with ZIKV resulted in a reduction in organoid size [17,25,37,38,39,40,41,42,43,44,45,46] and surface folding [47], which recapitulated microcephaly and lissencephaly, respectively, as is seen in affected children [48]. The reduced growth of ZIKV infected organoids resulted from a disruption in the cellular organization of the organoid [49,50,51,52]. ZIKV infected organoids had fewer and smaller ventricles and ventricular zones, with fewer NPC compared to non-infected organoids [37,38,40,44,50,52,53,54]. The cortical layer contained a reduced number of neurons [38]. Interestingly, one study reported increased neuron marker expression after infection, potentially due to the premature differentiation of NPCs [42]. Most studies consistently observed that NPCs located in the ventricular, subventricular, and intermediate zone were the main target of ZIKV infection [17,37,39,41,42,43,44,52]. However, when organoids were infected at later timepoints (56 to 119 days), neurons and astrocytes were also susceptible to ZIKV infection [17,40,55,56]. In the infected organoids, ZIKV caused a reduction in NPC proliferation [42,51] and an increase in cell death [17,42,50,51]. 

ZIKV-induced immune responses varied in brain organoids of different ages. Brain organoid age and cellular content had an influence on ZIKV-induced interferon type 1 (IFN-1) pathway activation. In 10 day old organoids, only modest IFN-1 activation was observed. Inducing the IFN-1 response via IFN-β treatment was neuroprotective, as it reduced ZIKV infection and subsequent pathogenic effects [37]. Similarly, treatment with 25HC, the enzymatic product of an IFN-β stimulated gene, protected against ZIKV infection [46]. In 10 day old cerebral organoids, ZIKV-mediated microcephaly was dependent on Toll-like receptor 3 (TLR3) overactivation [39]. In contrast, in 17 day old forebrain dorsal organoids, the role of TLR3 in ZIKV pathogenesis could not be confirmed [51]. Instead, in these organoids, microcephaly resulted from IFN-independent IFN-stimulated gene (ISG) overactivation [51]. Potentially, the discrepancy between these results is due to the different cellular composition of these models [51]. In these studies, only the innate immune system was studied since cerebral organoids lack immune cells. To overcome this limitation, brain organoids were co-cultured with microglia, which, upon ZIKV infection, showed increased expression of *TNF-α, CCL2, interleukin 1 Beta* (*IL-1β*), and *IL-6* cytokine response compared to cerebral organoids without microglia [26]. The role of microglia in ZIKV infection was further studied in 75 day old organoids containing microglia. After ZIKV infection, microglia became activated, leading to upregulation of the complement pathway, IFN response, cytokine expression, and astrocytosis [25]. The activated microglia excessively phagocytosed synapses, demonstrating a potential harmful effect of microglia activation after ZIKV infection [25,57]. Finally, the neuroprotective or antiviral activity of betulunic acid, enoxacin, methylene blue, JMX0207 blue, sofosbuvir, and emricasan, against ZIKV, was confirmed in cerebral organoids [44,50,58,59,60,61]. Inhibition of *AXL*, a potential entry receptor of ZIKV identified in non CNS cells, did not mitigate ZIKV-induced pathogenic effects or pathogenesis infection in 24 day old cerebral organoids [43], indicating that AXL is not essential for infection. However, another study reported *AXL* upregulation in microglia, upon ZIKV infection, in microglia-containing 75 day old cerebral organoids [25], suggesting that AXL has a role in ZIKV infection. The discrepancy between these results is, most likely, because AXL is essential for infecting glial cells and not NPCs [62,63].

Taken together, these studies show that brain organoids recapitulate the microcephaly phenotypes observed in patients. Additionally, studies in brain organoids consistently described that, by infecting NPCs, ZIKV infection led to depletion of the progenitor pool through apoptosis or premature differentiation, which greatly inhibited neurogenesis, leading to the microcephaly phenotype [54]. These findings are consistent with studies in mice, where ZIKV targeted NPCs and caused microcephaly as well [64]. Lastly, the innate immune response upon ZIKV infection was not consistent across studies, which was most likely due to variation in organoid age and cellular composition. Further studies will have to determine whether boosting an attenuated immune response can protect the developing brain from microcephaly or if the immune response is overactivated and needs to be inhibited to prevent neurotoxicity. Such further studies would need to include microglia, as they are important players in the antiviral immune response of the CNS. 

#### 3.2.2. SARS-CoV-2

SARS-CoV-2 is an enveloped, single-stranded RNA virus, belonging to the *Coronaviridae* family [65]. Neurological symptoms and sequelae, during or after SARS-CoV-2 infection, have been reported [66,67]. Whether SARS-CoV-2 infection causes these complications through direct infection of the CNS or via a SARS-CoV-2-induced immune response is still unclear [65,68]. To investigate if the CNS is susceptible to direct infection, 8 out of 11 studies on SARS-CoV-2 (73%) reported on SARS-CoV-2 receptor expression in the CNS (Table 1). In vivo, the SARS-CoV-2 receptor, angiotensin-converting enzyme 2 (ACE2), is expressed in the human brain, especially in the ChP, an epithelial cell layer that lines the ventricles and produces cerebrospinal fluid (CSF) [69]. *ACE2* in the CNS is expressed at lower levels in the CNS compared to the lungs [69]. Similar to in vivo findings, *ACE2* expression in cerebral organoids were reported [10,16,54,70,71,72] at low levels compared to human respiratory epithelial cells [54] or lung organoids [72]. *ACE2* was reported to be expressed in neurons and astrocytes [72,73]; however, expression levels were lower compared to cells of choroid plexus regions [10,16]. Interestingly, one study reports a trend towards increased *ACE2* expression upon SARS-CoV-2 infection [74]. Besides ACE2, transmembrane serine protease 2 (TMPRSS2) is another entry factor for SARS-CoV-2. *TMPRS2* mRNA was either expressed below the detection limit [70] or at low levels, depending on the brain organoid model and age [10,16,71,72]. Similar to *ACE2*, *TMPRSS2* was predominantly expressed in choroid plexus clusters [16]. *TMPRSS2* was also expressed in the neurons, but not in the astrocytes, of cortical organoids [16,72]. A third host factor that is important for SARS-CoV-2 infection is Neuropilin-1 (NRP1). *NRP1* was expressed in choroid plexus, hippocampal, and cortical organoids [10,71,72]. 

Drastic cytopathic effects and morphological changes were not reported for SARS-CoV-2 infection in brain organoids. Cell death was reported in 5 out of 11 papers (45%), in both infected cells and bystander cells, in the region of infection [10,70,74,75,76,77]. Further reported pathogenic effects of SARS-CoV-2 infection were cell fusion [10,77], Tau hyperphosphorylation [75], and upregulation of metabolic processes, leading to a hypoxic environment [71]. More pronounced cytopathic effects were observed in infected ChP organoids; the barrier function of the ChP was impaired through the downregulation of tight junctions, and secretory functions were compromised [10,16,74]. 

A total of 10 out of 11 studies reported results on cell tropism of SARS-CoV-2, with conflicting results. For instance, of the 10 studies that investigated whether neurons are susceptible to SARS-CoV-2, six studies reported infection [70,71,73,75,76,77], while in four studies, neurons were reported to support little-to-no infection of SARS-CoV-2 [10,16,29,74]. These studies were performed in cerebral and brain-region-specific organoids, ranging from 15 to 180 days old, hampering the comparison of the results on susceptibility of neurons to SARS-CoV-2 infection. Three studies reported on the susceptibility of NPC to SARS-CoV-2 infection [71,74,77]. Studies using 35 and 60 day old cortical organoids reported infection of NPCs [71,77], while 180 day old cortical organoids demonstrated little-to-no infection of NPCs [74]. Astrocytes were investigated three times and were reported to be infected in all studies [10,29,74], although, in one, the infection of astrocytes was sparse [10]. ChP epithelial cells were studied in three studies and displayed infection in all reports, and each of these studies claimed that SARS-COV-2 preferentially infected ChP cells over neurons [10,16,74], which was congruent with the beforementioned higher expression of SARS-CoV-2 receptors in ChP cells, as compared to neurons [10,16]. Infection of neural-perivascular assembloids, a model of the neurovascular unit, showed that pericytes were also susceptible for SARS-CoV-2, which supported the SARS-CoV-2 spread to astrocytes, presenting a potential route of infection of the CNS [29]. Overall, conflicting results were described in tropism studies of SARS-CoV-2, which could be the result of differences in brain organoid models and organoid age at time of infection, since organoid age influences the cellular composition of brain organoids and the maturation status of cell types [74]. 

Regarding inflammatory response, ChP organoids showed an increase in inflammatory cytokines *CCL7*, *interleukin-32* (*IL-32*), *CCL2*, *MCP1*, *IL-18*, and *IL-8* upon infection [10]. Cerebral organoids, infected with SARS-CoV-2, showed no IFN or IFN-stimulated gene signatures [71], while assembloids of cerebral organoids with pericyte-like cells elicited an astrocyte-mediated IFN-1 response after SARS-CoV-2 infection [29].

In summary, organoids expressed ACE2, TMPRSS2, and NRP1, mainly by ChP cells, similar to expression in human brains in vivo. Accordingly, ChP cells were more susceptible to SARS-CoV-2 infection compared to neurons, NPCs, and astrocytes. Infection of neurons was debatable, but it led to cell death, while infection of ChP cells led to a loss of barrier function and cytokine release. Whether SARS-CoV-2 directly infects the CNS in vivo remains to be elucidated, but these studies in brain organoids can help with understanding which cells in the CNS are susceptible to infection and which potential pathogenic effects could result from CNS infection. 

#### 3.2.3. HSV1

HSV1 is an enveloped double-stranded DNA virus of the *Herpesviridae* family. HSV1 is the most common cause of encephalitis [7]. In the absence of antiviral therapy, neonatal HSV1 CNS disease causes high mortality, with long-term neurological sequelae [78]. HSV1 infection of the CNS has been investigated in four studies (Table 1). In two publications, infections were performed on iPSC-derived immature cerebral organoids at 10, 15, and 45 days in culture [37,79], while in the other two studies, infections were performed on mature 70 day old organoids generated from iPSC-derived NPCs [7,80]. Both cultures were susceptible for HSV1 infection. The studies on mature organoids reported infection of neurons [7,80], while in immature cerebral organoids, NPCs were infected [37]. The maturity of the organoid also affected the cytopathic effect. Infection of immature organoids led to drastic changes in organoid architecture and reduction in size, most likely due to impaired neurogenesis caused by the infection of NPCs [37,79]. These effects on organoid morphology and size were not reported in studies of mature organoids, in which neurons (and not NPCs) were infected. In these mature organoids, reactivation of latent HSV1 infection in neurons led to degeneration of neuronal processes and cell-cell fusion, followed by generation of neuronal syncytia. However, HSV1 reactivation occurred infrequently (in 4 out of 23 organoids), which is in contrast to consistent HSV1 reactivation in 2D neuronal cultures, highlighting differences between 2D and 3D cultures [7].

HSV1 infected organoids responded with poor activation of ISGs [37]. Most cells lacked IFN-1 and ISG induction, and both *IFN-α2* and *IFN-β* expression were unchanged [37]. While IFN-α2 treatment could prevent growth defects in infected organoids, IFN-β treatment did not prevent cytopathic effects, as IFN-β stimulated ISGs were inhibited by viral protein ICP34.5 [37]. Furthermore, HSV1 infection led to the activation of astrocytes, as well as the abnormal proliferation and activation of microglia in the organoids [79]. These cellular responses were linked to neurodevelopmental disorders in vivo [79]. Together, these studies showed that, in immature organoids, HSV1 infection led to the disruption of fetal brain development, with associated organoid size reduction, and suppressed IFN responses.

#### 3.2.4. HCMV

HCMV is a large, enveloped double stranded DNA virus, and like HSV, it is a member of the *Herpesviridae* family. Congenital HCMV infection can lead to a variety of CNS disorders, such as hearing loss, cognitive impairment, and microcephaly [81]. All four included publications on HCMV reported on the capability of HCMV to infect brain organoids (Table 1). HCMV infection was only observed in a small subset of cells [37,82]. Similar to HSV, HCMV infected NPCs in both in 10 and 45 day old brain organoids [37,83]. Whether HCMV infection resulted in reduced organoid growth was dependent on brain organoid age. Organoids infected at the iPSC stage (day 0) and differentiated for 52 days showed no growth attenuation, just as with 10 day old infected organoids [37,84]. Infection of 45 day old organoids, however, did reduce organoid size, due to reduction in proliferation and increased cell death [83]. Irrespective of organoid age, HCMV infection disrupted organoid morphology. HCMV infection before differentiation, at the iPSC stage, led to a reduction in cortical structures and thin, underdeveloped multilayer areas. Areas of necrosis, which contained vacuoles within the cortical structures, were observed [84]. Infection of 30 and 60 days old cortical organoids did not affect NPC marker expression. However, NPC organization in neural rosettes was abolished by HCMV infection [82]. The misplacement of NPCs probably inhibited neurogenesis, since HCMV infected organoids contained less terminally differentiated neurons compared to mock infected organoids [82]. Organoids infected after 45 days presented thin subventricular zones, as well as cortical layers [83]. 

Besides effects on organoid size and cellular organization, HCMV infection also affected cellular function. HCMV infection resulted in the downregulation of genes involved in calcium signaling and the disruption of calcium function in 30, 45, and 60 day old organoids [82,83]. Calcium signaling is critical for NPC proliferation, migration, and differentiation and may, thus, be involved in the impaired neurogenesis caused by HCMV infection [82]. Additionally, neural network activity was reduced after HCMV infection [83]. These phenotypes were rescued upon treatment with neutralizing antibodies, thus proposing a potential treatment for HCMV infections [83]. Treatment with antiviral maribavir, which inhibited HCMV infection in 2D-NPCs, did not limit infection in cortical organoids [82]. 

In summary, even though only a subset of cells, mostly NPCs, were infected by HCMV, organoid organization and cellular function were disrupted, which could be prevented by neutralizing antibodies.

#### 3.2.5. DENV

DENV, as with ZIKV and JEV, is a member of the *Flaviviridae* family. DENV is a single positive stranded RNA virus, and it is transmitted through mosquitos. DENV is not considered neurotropic, but neurological signs are reported at incidences of 0.5 to 20% [85]. Symptomatic DENV infection during pregnancy is associated with an increase in congenital malformations of the brain but not at the high frequencies, as seen with congenital ZIKV infections [86]. Therefore, in studies that reported on DENV infection of brain organoids (Table 1), DENV was used as negative control for ZIKV infection-induced pathologies [26,45,47,87]. Brain organoids were susceptible to DENV infection to a lesser extent than to ZIKV and irrespective of the presence of microglia [47]. While ZIKV infection of brain organoids led to inhibition in cell proliferation, cell death, and reduced organoid growth, DENV did not cause these pathogenic effects [45,47,87]. Still, DENV infection led to upregulation of *TNFα, CCL2, IL-1β,* and *IL-6* [26]. The lack of severe effects of DENV infection in brain organoids, compared to the infection of related flaviviruses, shows that brain organoids model virus-specific pathogenesis. 

#### 3.2.6. Brain Organoid Studies on AAV, MeV, Rabies Virus, JEV and LACV

Brain organoids have been used to study AAV, MeV, Rabies virus, JEV, and LACV. As there have been fewer than four studies published per virus, the finding of the studies are briefly discussed below. 

AAV infection of brain organoids was studied in three reports [88,89,90]. AAV differs from the other reported viruses since it is does not cause CNS disease but, instead, is used as a vehicle in gene therapy [91]. Brain organoids were useful in gene therapy development, as they were used for investigating tropism of AAV, comparing transduction efficiency of AAV serotypes, and demonstrating that AAV effectively delivered treatment against neurodegenerative diseases [88,89,90].

MeV, a highly contagious respiratory RNA virus belonging to the *Paramyxoviridae* [92], which can infect the CNS in rare cases, was evaluated in one study. The main finding of this study was that the cerebral organoid microenvironment, which positively selected mutations that enable MeV to fuse membranes without receptor binding, led to an increased spread of infection in cerebral organoids. This increased spread of the MeV mutant triggered a strong IFN-1 response, which was not able to prevent the spread of MeV [93]. Brain organoids were infected with rabies virus in one study; however, the goal of this study was to demonstrate a virus stamping technique and not to study rabies virus pathogenesis [94]. Chikungunya virus, an RNA virus from the *Togaviridae* family, was studied in cerebral organoids derived from Parkinson’s disease (PD) iPSCs and non-PD iPSC derived cerebral organoids. The immune response of PD cerebral organoids was altered compared to the non-PD organoids, highlighting the need for studies on the influence of host genetic variation on host-pathogen interactions [95]. John Cunningham virus (JCV), a DNA polyomavirus, was studied once in cerebral organoids [96]. JCV causes opportunistic infections in immunocompromised individuals, leading to progressive multifocal leukoencephalitis, a fatal demyelinating disease [97,98]. Brain organoids have been shown to be a suitable model for JCV pathogenesis with JCV infection of brain organoids, resulting in viral replication and infection of astrocytes and oligodendrocytes, while neurons were not infected [96].

JEV, which causes cytokine triggered neuroinflammation in young children, was investigated in a single study [99]. The study used telencephalon cortical organoids to show that, in immature organoids, JEV widely infected NPCs and oRG, while in mature organoids, mainly astrocytes are infected [99]. Since oRG are only sparsely present in rodents, the identification of oRG cells as targets of JEV infection highlights the importance of the human origin of these organoids [13]. Immature organoids were more susceptible to JEV infection, correlating with severe cytopathic effects, resulting in growth reduction. Most likely, effective antiviral immune response in mature organoids protected against JEV replication and cytopathic effects, while immature organoids had underdeveloped innate immune responses [99]. 

Finally, two studies reported on LACV, an orthobunyavirus and a common cause of pediatric encephalitis in the United States of America [100]. LACV infection of cortical organoids reduced metabolism and loss of cytoarchitecture. A robust IFN response protected LACV-infected NPCs from cell death, while both infected and adjacent neurons underwent apoptosis [101]. Treatment of 3 and 11 week old cortical organoids with rottlerin inhibited LACV replication and limited virus induced reduction in organoid viability [102]. 

In summary, the limited number of studies on these viruses evaluated the therapeutic efficacy of AAV, the evolution of neurotropism of MeV, and the role of the innate immune response in an infection of the brain by JEV and LACV. These studies provide the first basis for the use of brain organoids to study these viruses.

## 4. Discussion

In this review, we summarized how brain organoids are used as models for studying viral infections in the CNS, with particular focus on the organoid morphology, viral tropism, organoid age at time of infection, and immune responses of virus-infected brain organoids. Here, we discuss the benefits and limitations of using brain organoid models for virology studies.

From this literature review, we found that ZIKV and SARS-CoV-2 are the most studied viruses in brain organoid models. Leaving other common viral causes of CNS disease understudied in brain organoids. A likely explanation is that ZIKV and SARS-CoV-2 caused sudden major outbreaks, which boosted research worldwide. This urgency, combined with the lack of suitable animal models, created an impulse in research using human brain organoids. Next, we described a great variation in the ages of brain organoids at the time of viral infection. Since brain organoids differentiate over time, and specific cell types only develop at certain stages, the age of the brain organoid dictates its cellular composition. Moreover, the maturation status, the receptor expression, and the innate immune responses change with age [37], influencing the tropism, pathogenic effect, and immune response. The use of brain organoids of various ages for the studies, hampered a comparison of results and could explain the heterogenicity of the results. It was notable that not every study clearly reported on the age of the brain organoid at the time of infection or gave a rationale for the chosen organoid age. This is an important limitation of these studies, as we observed that the brain organoid age, at time of infection, influenced the tropism for ZIKV, SARS-CoV-2, HSV1, and JEV, as well as the severity of the pathogenic effect of the viral infection. As brain organoids differentiate over time, they allow for the study of the effects of viral infection on the developing brain. This is very informative for research on viruses that are known to cause neuronal complications in the developing brain (of young patients), such as ZIKV, HCMV, JEV, and HSV1. For example, to observe effects of ZIKV infection on neurodevelopment, brain organoids were infected at early stages of maturation, while studies on SARS-CoV-2 typically used brain organoids that, on average, have matured for a longer period. 

One of the great benefits of brain organoids, as a model for neurotropic viruses, is that brain organoids consist of a mixture of cell types, which are organized similarly to the human brain. As such, they were shown to be suitable for investigating cell tropism in combination with the expression of receptors necessary for viral entry in viral infection of the brain. Besides, having multiple cell types in one culture influences the immune response, as was demonstrated when brain organoids were complemented with microglia and infected with ZIKV. Additionally, by being grown in 3D, brain organoids have different gene expression profiles [103], stiffness, and cell polarity, when compared to neuronal cultures grown in 2D, making for a more physiological human brain model. This resemblance allows for more in-depth studies on cell function and host-pathogen interaction studies. For example, ZIKV and HSV1 infection led to stronger inhibition of IFN-1 responses in a 3D versus a 2D model [37]. Furthermore, the 3D structure enabled the study of morphological changes in the organoids that go unnoticed in a 2D-culture. For instance, in HCMV infected organoids, NPC marker expression was not reduced. However, the cellular organization of the brain organoid was affected, which inhibited neurogenesis. Another example is the observed reduction in size of ZIKV, HSV1, and HCMV-infected organoids that recapitulate the microcephaly phenotype that is presented in congenital cases. A further benefit of the 3D structure in virus studies is that pathogenic effects of infected cells on uninfected cells became apparent. We described multiple examples of cell death in bystander cells. These effects are unlikely to be seen in 2D models, since excreted factors from infected cells are diluted in the growth medium, while in a 3D structure, these can accumulate in the microenvironment. The 3D structure is also important for testing antiviral therapeutic candidates, since the cell density of 3D organoids is more similar to in vivo tissue, which affects diffusion of the antiviral compound. This could be the explanation of why maribavir was effective in preventing HMCV infection in 2D neuronal cultures but not in 3D cortical organoids. Finally, for testing antiviral compounds that reduce viral replication by targeting host factors, the human origin of brain organoids is of additional benefit. 

Besides these advantages, human brain organoid technology still has limitations. First, both immature and mature brain organoids have a gene expression pattern of a fetal human brain and, as such, have a fetal identity [20,103]. This is an advantage when studying viruses that cause congenital neurological diseases during fetal neurodevelopment. However, the extrapolation of results from viral studies in brain organoids to adult human viral brain infection is less evident than the extrapolation of results to infant or child human viral brain infections. Second, in general, brain organoids do not contain microglia, the resident immune cells of the CNS, because microglia originate from a different germ layer than the neural tissue [22,23]. Microglia have an important role in brain development, viral spread, immune response activation, and virus-mediated pathology in the brain [57,104,105]. However, it is possible to generate brain organoids that contain microglia [22,79], or to generate so-called complex brain organoid models, by incorporating immune cells into a co-culture [25,26], which allows for the study of the role of microglia in viral pathogenesis. Without the cellular immune system, brain organoids still give insight into the innate immune response, as demonstrated by the many studies that investigated both neuroprotective and neurotoxic effects of the IFN response, how viruses were able to inhibit the IFN response, and whether inducing or inhibiting the IFN response has therapeutic potential. However, since microglia influenced the IFN response upon ZIKV infection, future studies on the immune response against viral infection should consider using brain organoids with incorporated microglia. A third limitation of brain organoids is the lack of a (BBB), which hampers studies on neuroinvasion. This is a main limitation for studies on SARS-CoV-2 infection, as it is unknown whether neurological complications in SARS-CoV-2 patients are caused by direct infection of the CNS or via a SARS-CoV-2-induced immune response. The clinical relevance of the infection of the ChP by SARS-CoV-2, therefore, remains unclear as long as neuroinvasion has not been proven. To investigate the neuroinvasive potential of SARS-CoV-2, neural-perivascular assembloids containing pericytes, which is one of the cellular components of the BBB, were used for SARS-CoV-2 studies [29]. Currently, BBB models are being developed that could be combined with brain organoids [106] and further enable the study of neuroinvasion. A last limitation is the lack of standardization. We already discussed the variation in organoid age at time of infection and its effect on the heterogeneity of results between studies. Additionally, the diversity in protocols, for both unguided whole brain and guided brain-region specific organoid generation, could be another cause of heterogenic results. However, both whole brain and region-specific organoid models have a role in generating and testing hypotheses, with region-specific organoid models being best applied to testing-specific hypotheses [18]. For instance, in whole brain organoids, SARS-CoV-2 was found to mainly infect ChP cell clusters, leading to the hypothesis that SARS-CoV-2 disrupts the ChP barrier, which was tested by subsequent studies in ChP organoids. 

In this review, we analyzed studies on different viruses using brain organoids. There are still other neurotropic viruses that could benefit from the application of brain organoids, such as emerging virus infections with West Nile Virus, Varicella Zoster Virus, the picornaviruses parechovirus A3, or enterovirus D68. Our inclusion criteria might have excluded valuable articles that did not comply with the inclusion criterium of a pluripotent stem cell origin of the brain organoid model [107]. Therefore, we missed other important applications of organoids in virology—for instance, their applicability to study human immune deficiency virus (HIV) [107].

In conclusion, brain organoids are a versatile model to study multiple aspects of viral pathogenesis and therapeutic testing in the CNS. The human origin, content of different cell types, and 3D structure of brain organoids give important benefits for studying neurotropic viruses. Organoid models can be tailored to the research question by using region-specific organoids or adapting the time of infection. Brain organoids have been used to study different viruses and have recapitulated virus-specific cytopathic effects. With the expansion of this field of research, standardization of protocols is needed to reduce heterogeneity of results between studies. Since brain organoids are not a complete model of the CNS, brain organoid technology still needs to be optimized by incorporating adjacent cells, such as microglia and endothelial cells [108]. This increase in the complexity of brain organoid models will further improve the translation of results from virus research, using human brain organoids, to virus infection in a human being.

## Figures and Tables

**Table 1:** Research topics on brain organoid viral infection, depicted as percentage of studies that have reported on these different research subjects.

Virus	ZIKV	SARS-CoV-2	DENV	HSV1	HCMV	All Viruses
Viral Infection	27/30 (90%)	11/11 (100%)	2/4 (50%)	4/4 (100%)	4/4 (100%)	57/63 (90%)
Receptor expression	2/30 (7%)	8/11 (73%)	0/4 (0%)	0/4 (0%)	1/4 (25%)	11/63 (17%)
Organoid organization and size	21/30 (70%)	3/11 (27%)	3/4 (75%)	3/4 (75%)	1/4 (25%)	37/63 (59%)
Viral tropism	14/30 (47%)	10/11 (91%)	1/4 (25%)	3/4 (75%)	2/4 (50%)	34/63 (54%)
Cytopathic effect	17/30 (57%)	5/11 (45%)	2/4 (50%)	1/4 (25%)	1/4 (25%)	28/63 (44%)
Effect of infection on proliferation	7/30 (23%)	1/11 (9%)	2/4 (50%)	0/4 (0%)	1/4 (25%)	12/63 (19%)
Antiviral innate Immune response	7/30 (23%)	3/11 (27%)	1/4 (25%)	3/4 (75%)	0/0 (0%)	17/63 (27%)
Gene expression profile	4/30 (13%)	4/11 (36%)	0/4 (0%)	1/4 (25%)	1/4 (25%)	14/63 (22%)
Antiviral drug testing	12/30 (40%)	0/11 (0%)	0/4 (0%)	0/4 (25%)	2/4 (50%)	18/63 (29%)

**Table 2:** Main findings of studies on ZIKV, SARS-CoV-2, HSV1, HCMV, and DENV using brain organoids.

Virus	Main Finding in Brain Organoids
ZIKV	Infection of NPCs led to impaired neurogenesis and microcephaly. Innate immune response was inconsistent across studies. Microglia induced immune response upon ZIKV infection.
SARS-CoV-2	SARS-CoV-2 receptors expressed predominantly in ChP cells. ChP cells were more susceptible compared to neurons. Infection of ChP impaired barrier function and induced cytokine release.
HSV1	HSV1 infection disrupted brain organoid development. HSV1 reactivation led to neuronal cellular pathologies, but occurred infrequent. HSV1 inhibited IFN response.
HCMV	HCMV infection of a subset of NPCs disrupted organoid organization and cellular function. HCMV infection reduced growth dependent on organoid age Neutralizing antibodies but not antiviral maribavir prevented disease phenotype.
DENV	DENV infection did not lead to severe pathogenic effects such as related flavivirus ZIKV.

## Supplementary Materials

The following supporting information can be downloaded at: https://www.mdpi.com/article/10.3390/v14030634/s1, Table S1: List of included studies.
