# Supplementary material for: Human Brain Organoids as Models for Central Nervous System Viral Infection
Source: Viruses. 2022 Mar 18;14(3):634. doi: 10.3390/v14030634 (PMC8948955; doi:10.3390/v14030634)
Supplement: Supplementary file 1 [file viruses-14-00634-s001.zip › viruses-1605808-supplementary.pdf]

Study	publication year	virus studied	Viral infection	Receptor expression	Organoid organization & size	Viral tropism	Cytopathic effect	Effect on proliferation	Innate immune response	Gene expression profile	Antiviral drug testing	organoid age (days)
Cugola, F.R. [53]	2016	ZIKV	■				■	■			■	n.r.
Dang, J. [39]	2016	ZIKV	■		■	■			■			10
Garcez, P. P. [45]	2016	DENV			■							35
Garcez, P. P. [45]	2016	ZIKV			■							35
Qian, X. [40]	2016	ZIKV	■		■	■	■	■				14,28,80
Wells, M.F. [43]	2016	ZIKV	■		■	■	■				■	24
Xu, M. [61]	2016	ZIKV	■				■				■	18
Gabriel, E. [54]	2017	ZIKV	■		■	■						9
Li, Y. [47]	2017	DENV	■		■		■					19
Li, Y. [47]	2017	ZIKV	■		■		■	■				19,30
Li, C. [46]	2017	ZIKV	■						■		■	20
Sacramento, C. [60]	2017	ZIKV	■								■	n.r.
Salick, M. R. [41]	2017	ZIKV	■		■	■	■					24
Watanabe, M. [17]	2017	ZIKV	■	■	■	■	■		■	■	■	21, 56
Yoon, K. J. [87]	2017	DENV			■			■				45
Yoon, K. J. [87]	2017	ZIKV			■			■				45
Zhou, T. [52]	2017	ZIKV	■		■	■	■	■			■	20
Abreu, C. M. [26]	2018	DENV	■			■	■	■	■			56
Abreu, C. M. [26]	2018	ZIKV	■			■	■	■	■			56
Janssens, S. [55]	2018	ZIKV	■			■				■		119
Kunze, C. [88]	2018	AAV	■			■					■	n.r.
Schubert, R. [94]	2018	Rabies	■									n.r.
Zhang, B. [99]	2018	JEV	■		■	■	■	■	■			24,55,10
Ayala-Nunez, N. V.	2019	ZIKV	■									21,35
Brown, R. M. [84]	2019	HCMV	■		■							0
D'aiuto, L. [7]	2019	HSV1	■		■	■						70
Janssens, S. [56]	2019	ZIKV	■			■						84
Latour, Y. L. [90]	2019	AAV									■	70
Liu, L. [51]	2019	ZIKV	■		■		■	■	■	■	■	17,23
Setoh, Y. X. [42]	2019	ZIKV	■		■	■	■					15
Sison, S. L. [82]	2019	HCMV	■		■						■	30,60
Winkler, C. W. [101]	2019	LACV	■		■	■	■		■	■		n.r.
Xu, Y. P. [44]	2019	ZIKV	■		■	■						20,38
Abrahams, E. E. [80]	2020	HSV1	■			■			■			84
Albanese, A. [38]	2020	ZIKV	■		■		■					21

Bullen, C. K. [70]	2020	SARS-CoV-2	■	■		■	■					n.r.
Cavalcante, B. R. R. [50]	2020	ZIKV	■		■		■					42
Depla, J. A. [89]	2020	AAV	■								■	66
Jacob, F. [10]	2020	SARS-CoV-2	■	■		■	■		■	■		47,67
Li, Z. [58]	2020	ZIKV	■		■						■	21
Li, Z. [58]	2020	ZIKV	■		■						■	22
Long, R. K. M.	2020	ZIKV			■							63
Pedrosa, C. D. S. G.	2020	ZIKV	■			■	■					50
Pellegrini, L. [16]	2020	SARS-CoV-2	■	■	■	■				■		55
Pettke, A. [49]	2020	ZIKV	■		■		■				■	14,15
Qiao, H. [79]	2020	HSV1	■		■				■			15,45
Ramani, A. [75]	2020	SARS-CoV-2	■	■		■	■					15,60
Sun, G. [83]	2020	HCMV	■	■	■	■	■	■		■	■	45
Yi, S. A. [73]	2020	SARS-CoV-2	■	■		■						180
Zhang, B. Z. [77]	2020	SARS-CoV-2	■			■						35
Barreras, P. [96]	2021	JCV	■			■						35
Krenn, V. [37]	2021	HCMV	■		■	■						10
Krenn, V. [37]	2021	HSV1	■		■	■	■		■	■	■	10,40
Krenn, V. [37]	2021	ZIKV	■		■	■	■		■	■	■	10
Mathieu, C. [93]	2021	MeV	■						■	■	■	90
McMahon, C. L. [74]	2021	SARS-CoV-2	■	■	■	■						180
Ojha, D. [102]	2021	LACV	■								■	21, 77
Schultz, E. [95]	2021	CHIKV	■		■				■	■		53
Song, E. [71]	2021	SARS-CoV-2	■	■		■	■		■	■		60
Tiwari, S. K. [72]	2021	SARS-CoV-2	■	■								80
Wang, C. [76]	2021	SARS-CoV-2	■			■						60
Wang, L. [29]	2021	SARS-CoV-2	■		■	■	■	■	■	■		74
Xu, R. [25]	2021	ZIKV	■	■	■		■		■			75

Supplementary Table 1.

List of all included studies. The cells of the table are black if a study includes the topic and white if they do not report on it. Organoid age in days at time of infection is also depicted.
